# Prevalence and Predictors of Atherogenic Serum Lipoprotein Dyslipidemia in Women with Obstructive Sleep Apnea

**DOI:** 10.1038/srep41687

**Published:** 2017-01-30

**Authors:** Yunyan Xia, Yiqun Fu, Yuyu Wang, Yingjun Qian, Xinyi Li, Huajun Xu, Jianyin Zou, Jian Guan, Hongliang Yi, Lili Meng, Xulan Tang, Huaming Zhu, Dongzhen Yu, Huiqun Zhou, Kaiming Su, Shankai Yin

**Affiliations:** 1 Department of Otolaryngology Head and Neck Surgery & Center of Sleep Medicine, Shanghai Jiao Tong University Affiliated Sixth People’s Hospital, Yishan Road 600, Shanghai, 200233, China; 2 Otolaryngological Institute of Shanghai Jiao Tong University, Yishan Road 600, Shanghai, 200233, China; 3 Clinical Research Center, Shanghai Jiao Tong University School of Medicine, South Chongqing Road 225, Shanghai, 200020, China

## Abstract

Obstructive sleep apnea (OSA) is associated with dyslipidemia. However, no study has focused on dyslipidemia in women with OSA. The aim of this study was to determine the prevalence and risk factors for dyslipidemia in women with OSA. Between 2007 and 2013, 570 eligible female patients with suspected OSA were consecutively recruited. The analyzed data consisted of polysomnography parameters, biochemical indicators, and anthropometric measurements. Serum lipid levels and dyslipidemia were compared. Binary logistic regression and multivariate linear regression models were used to determine the independent risk factors influencing serum lipids. After multivariate adjustment, there were essentially no major differences in serum lipid levels among patients with no to mild, moderate, and severe OSA nor did serum lipid levels change with OSA severity. Dyslipidemia in total cholesterol, triglycerides, low-density lipoprotein cholesterol, apolipoproteins(apo) B and apoE increased with OSA severity, but only in non-obese subjects and those <55 years of age. Age, body mass index, waist to hip ratio, glucose and insulin were major risk factors for most serum lipids after multivariate adjustments. Our results indicate that, in women with OSA, age, obesity/central obesity, and insulin resistance are major determinants of dyslipidemia.

Obstructive sleep apnea (OSA) is one of the most common sleep disturbances, affecting approximately 4% of male, and 2% of female, middle-aged adults^1^. The prevalence of OSA has increased as the rates of obesity have increased in the general population^2^. Multiple clinical studies and meta-analyses showed that OSA is associated with higher total cholesterol (TC), triglyceride (TG), and low-density lipoprotein cholesterol (LDL-C), and lower high-density lipoprotein cholesterol (HDL-C)^3^,^4^,^5^,^6^,^7^. Meanwhile, higher LDL-C and TG, and lower HDL-C, were found to be independent risk factors for OSA^8^,^9^. A nonlinear dose-effect relationship between dyslipidemia and OSA severity was recently established^8^,^9^.

Although men are twice as likely as women to have OSA, it is associated with left ventricular hypertrophy, incident heart failure, or even death in women but not in men^10^. In addition, women with OSA have poorer survival rates than men with OSA^11^. Thus, OSA in women should not be ignored. Different from men, women experience a unique menopausal transition. Menopause, along with age and obesity, significantly affects the occurrence and severity of OSA^12^,^13^,^14^. The incidence of OSA is significantly higher in postmenopausal women than premenopausal women^15^. One previous study showed that the blood pressure profile in postmenopausal women with OSA was mainly affected by body mass index (BMI) and the apnea-hypopnea index (AHI), while in premenopausal women, it was predominantly associated with BMI^16^. Similarly, we hypothesized that, among OSA patients, different unique factors influence lipid profiles across distinct age groups and obesity stages.

To date, no study has explored the relationship between OSA and dyslipidemia in women. To address this evidence gap, we performed a large cross-sectional study focusing on the prevalence of atherogenic serum lipoprotein dyslipidemia, and exploring the predictors of dyslipidemia in women with OSA.

## Results

### Characteristics, serum lipid levels, and prevalence of lipid abnormalities in female OSA patients stratified by disease severity, obesity, and age

**Figure 1:**
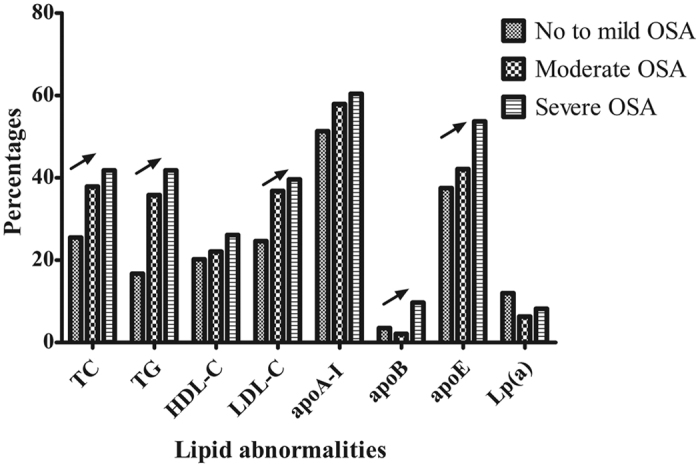
Prevalence of lipid abnormalities in women according to OSA severity (n = 570). Abbreviations: OSA, obstructive sleep apnea; TC, total cholesterol; TG, triglycerides; HDL-C, high-density lipoprotein cholesterol; LDL-C, low-density lipoprotein cholesterol; apo, apolipoprotein; Lp(a), lipoprotein(a).

The 570 female participants were divided into three groups: no to mild OSA (n = 341), moderate OSA (n = 95), and severe OSA (n = 134). After adjustments for age, BMI, waist to hip ratio (WHR), serum insulin levels, and glucose levels, there were no significant differences in serum lipid levels (except lipoprotein (a)) among the three groups (all p > 0.05). Bootstrap analysis showed that most of the p values generated from the repeated analysis of covariance(ANCOVA) were >0.05 and it confirmed the absence of significant difference in serum lipid levels (Supplementary Figs 1–3). In addition, after multivariate adjustment and with the exception of apolipoprotein(apo)B, there were no increases or decreases in serum lipid level that corresponded to increasing OSA severity (linear trend, all p > 0.05; Table 1). The prevalence of dyslipidemia in TC, TG, LDL-C, apoB, and apoE increased with increasing OSA severity (linear trends, p < 0.05) (Table 2, Fig. 1).

A subgroup analysis showed that serum lipid levels did not differ among the three groups nor were there corresponding increases or decreases in OSA severity, except with regard to apoB in obese subjects, TC and apoB in subjects <55 years of age, and apoA-I in subjects ≥55 years of age (Supplementary Table S1). The prevalence of dyslipidemia (including in TC, TG, LDL-C and apoB) increased with increasing OSA severity in non-obese subjects and those <55 years of age not in obese or older subjects (except apoA-I in older subjects) (Supplementary Table S2).

We also compared serum lipid levels and the prevalence of dyslipidemia across OSA severity, using a cut-off of 10 events/has a definition of OSA positivity^17^. At this cut-off value as well as at a cut-off of 5 events/h, our conclusions did not change (Supplementary Tables S3 and S4).

### Associations among age, obesity indices, sleep parameters, and natural log-transformed lipid levels in women

Binary logistic regression showed that age, WHR, and the oxygen desaturation index (ODI) (OR = 1.03, p < 0.01; OR = 1.80, p < 0.01; and OR = 1.02, p < 0.01, respectively) were independently associated with the presence of dyslipidemia after adjusting for BMI, insulin, glucose, the AHI, lowest oxygen saturation (LSpO_2_), and the micro-arousal index (MAI) (Table 3). By univariate regression analysis, all of the lipids were significantly associated with almost all of the selected factors (including age, BMI, WHR, insulin, glucose, AHI, ODI, MAI, and LSpO_2_; all p < 0.05; Supplementary Table S5). Multivariate regression showed that the independent factors were, for TC: age, WHR, and glucose level; for TG: age, WHR, and insulin level; for HDL-C: BMI and insulin level; for LDL-C: age and glucose level; for apoA-I: age and BMI; for apoB: age, WHR, glucose level, and LSpO_2_; and for apoE: age, WHR, and insulin level (Table 4). To further analyze the effects of age or menopausal status on lipids, the patients were divided into age ≤45 and age ≥55 groups, roughly corresponding to premenopausal and postmenopausal women, respectively^18^. After adjusting for confounding factors, the major independent determinants of dyslipidemia in premenopausal women were WHR and ODI (OR = 1.99, p < 0.01; OR = 1.02, p < 0.01, respectively), whereas in postmenopausal women only the MAI was identified (OR = 1.03, p < 0.01; Supplementary Tables S6 and 7).

## Discussion

This study showed that after adjusting for age, BMI, WHR, insulin, and glucose levels, there were, for the most part, no differences in the serum lipid levels among women with no to mild OSA, moderate OSA, and severe OSA. There was also no change in serum lipid levels with increasing OSA severity. Although prevalence of dyslipidemia increased with increasing OSA severity under the condition without multifactor adjustment (Fig. 1), subgroup analysis failed to demonstrate this trend in the prevalance of dyslipidemia among obese or older subjects. Multivariate regression analysis showed that age, WHR, and ODI were independently associated with dyslipidemia. Age, BMI, WHR, and insulin and glucose levels were major independent factors for almost all lipids, while sleep parameters were not.

The two previous studies that examined dyslipidemia in women reported a higher common lipid profile (i.e., TC, TG, HDL, and LDL) in women with than without OSA^19^,^20^. However, in our study, serum lipid levels did not significantly differ among the three OSA groups when adjusted for multiple confounding factors. In addition, the multivariate regression analysis showed that, with minor exception, none of the sleep parameters were independently associated with serum lipid levels. Thus, we conclude that OSA itself plays a limited role in dyslipidemia in women.

However, we did identify a positive independent association between age and TC, TG, LDL-C, and apoA-I, apoE, and apoB levels, consistent with the results of previous studies^21^,^22^,^23^. The unique features of the menopausal transition, characterized by fluctuating estrogen levels, may contribute substantially to serum lipid disorders. Previous studies showed a positive association between menopause and TC, TG, LDL-C, and apoB levels and between a lower HDL-C level and smaller HDL particle size, in analyses adjusting for age, BMI, and other potential confounders^21^,^22^,^24^. Among our patients, in those who were premenopausal women, the major independent determinants of dyslipidemia adjusted for confounding factors were WHR and ODI, while in postmenopausal women only the association with MAI was significant (Supplementary Tables S6 and 7).So, more importance should be attached on obesity in premenopausal women, and on OSA in postmenopausal women. Thus, future dyslipidemia studies should focus on different factors according to age and menopausal state.

Our study found that obesity/central obesity (BMI/WHR) was independently and positively associated with serum lipids in women with OSA, as also reported in some other studies^25^,^26^,^27^. Patients with OSA are typically more obese than non-OSA patients and are more likely to accumulate visceral adipose tissue (VAT) than BMI-matched controls^20^,^28^. VAT is more closely associated with a pro-atherogenic profile^29^,^30^ than subcutaneous adipose tissue (SAT), the significant effect of obesity/central obesity on serum lipids in our patients with OSA might have been due to greater VAT accumulation.

We also found that increased insulin resistance was independently associated with serum lipids. Insulin resistance increases insulin-stimulated hepatic lipogenesis and causes a general accumulation of ectopic lipids^31^,^32^. The intracellular accumulation of lipids then triggers trigger defects in insulin signaling and induces insulin resistance in muscle and liver^33^,^34^, initiating a vicious cycle. A study performed in overweight/obese patients with OSA showed that the pro-atherogenic lipoprotein abnormalities in OSA are associated with insulin resistance, but not with OSA severity or the degree of hypoxia^35^. Thus, glucose metabolism should be taken into account in analyses of lipid metabolism in women with OSA.

There were several limitations to our study. First, menopausal status and sex hormone levels were not determined and the actual effect of menopause on lipid metabolism therefore remains unclear. Second, despite adjusting for several common confounding factors, other, more complex factors, such as lifestyle, exercise status, and dietary habit, were not considered. Third, our study was observational and hospital-based rather than prospective and community-based design. Finally, the abnormalities in sleep architecture in our study may, at least in part, have been caused by the “first night effect,” during the single night of polysomnography(PSG) monitoring^36^,^37^. Despite these limitations, the sleep data and the relatively large sample size increased the credibility of our results.

In conclusion, in women with OSA, after multivariate adjustment there were almost no differences in serum lipid levels among those with no to mild OSA, moderate OSA, and severe OSA. Serum lipid levels also did not change with increasing OSA severity after multivariate adjustment. An increased prevalance of dyslipidemia with increasing OSA severity was seen only in non-obese subjects and those <55 years of age. Age, obesity/central obesity, and insulin resistance were major determinants of the levels of most of the analyzed lipids, after multivariate adjustment. Future studies should therefore focus on age, obesity/central obesity, and insulin resistance in women with OSA rather than on OSA itself in this group of patients.

## Materials and Methods

### Participants

**Figure 2:**
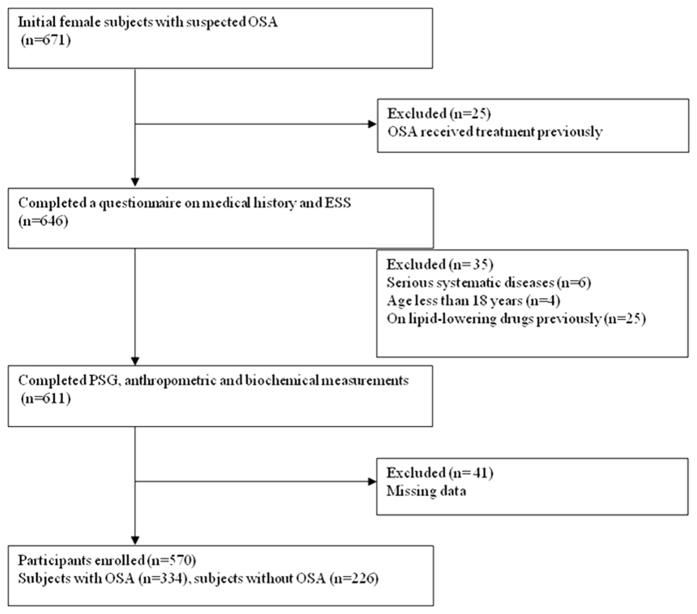
Enrollment flow chart for the study population. Abbreviations: OSA, obstructive sleep apnea; ESS, Epworth Sleepiness scale; PSG, polysomnography.

We consecutively enrolled 671 female subjects with suspected OSA, from January 2007 to July 2013, from the Shanghai Sleep Health Study (SSHS) sleep center. In total, 101 subjects were excluded for the following reasons: previously received treatment (e.g., weight loss surgery, Z-palatopharyngoplasty (ZPPP), or continuous positive airway pressure (CPAP)) (*n* = 25); presence of serious systematic diseases (e.g., heart failure or cerebrospinal fluid rhinorrhea) (*n* = 6), aged less than 18 years (*n* = 4); previously administered lipid-lowing drugs (*n* = 25); and missing data (*n* = 41). In total, 570 subjects were finally included in this study (Fig. 2).Written informed consent was obtained from each participant according to the guidelines of the National Ethics Regulation Committee. This study was approved by the Internal Review Board of the Institutional Ethics Committee of the Shanghai Jiao Tong University Affiliated Sixth Hospital, and was conducted in accordance with the Declaration of Helsinki.

### Overnight PSG parameters

Respiratory events were scored using laboratory-based PSG (Alice 4 or 5; Respironics, Pittsburgh, PA, USA). Electroencephalogram (EEG), electrooculogram (EOG), electrocardiogram (ECG), electromyogram (EMG), nasal and oral airflow, thoracic and abdominal respiratory effort, pulse oximetry, posture, and snoring data were obtained. The AHI was defined as the number of apnea and hypopnea events per hour during sleep. ODI was defined as the number of times per hour of sleep that the blood oxygen level dropped by ≥4% from baseline. MAI was defined as the number of arousals per hour of sleep. LSpO_2_ was defined as the lowest value of whole oxygen saturation observed during sleep. AHI was categorized as < 5, ≥5, ≥15, and ≥30 events per hour and represented no to mild OSA, moderate OSA, and severe OSA respectively, according to the American Academic Sleep Medicine criteria (AASM)^38^.

### Anthropometric measurements

Weight was measured using a weighing scale, with the subject standing still in light clothing and with shoes off. Height was measured as the maximum distance from the feet to the highest point on the head while standing with shoes off, feet together, arms by the sides, and heels, buttocks, and upper back in contact with the wall. BMI was calculated as: weight (kg)/height^2^ (m^2^). Neck circumference (NC) was measured at the level of the cricothyroid membrane while the subject was standing; waist circumference (WC) was measured midway between the lower costal margin and iliac crest; and hip circumference (HC) was measured as the maximum girth at the greater trochanters. The WHR was calculated as: WC (cm)/HC (cm). According to their BMI, patients were divided into obese (n = 120) and non-obese (n = 450) categories. According to the criteria in the *Guidelines for Prevention and Control of Overweight and Obesity in Chinese Adults*, obesity was defined as BMI ≥28 kg/m^2^ 39,40. Considering that most Chinese women experience menopause at 55 years old^18^,^41^, we also categorized participants by age: age <55 years (n = 382) and age ≥55 years (n = 118).

### Biochemical indicators

For each participant, a fasting blood sample was drawn from the antecubital vein. Serum lipid profiles (including TC, TG, HDL-C, LDL-C, apoA-I, apoB, apoE and Lp(a), fasting serum glucose, and fasting serum insulin were measured. Serum lipid profiles were measured in the hospital laboratory using routine procedures; serum glucose was measured using an H-7600 autoanalyzer (Hitachi, Tokyo, Japan), and serum insulin was measured using an immunoradiology method. Insulin resistance was calculated using HOMA-IR as: fasting serum insulin (μU/mL) × fasting plasma glucose (mmol/L)/22.5^42^. Dyslipidemia, in terms of TC, TG, HDL-C, LDL-C, apoA-I, apoB, apoE, and Lp(a), was defined as ≥5.17 mmol/L, ≥1.7 mmol/L, <1.03 mmol/L, ≥3.33 mmol/L, <1.2 g/L, >1.1 g/L, >0.05 g/Lor <0.03 g/L, and ≥0.3 g/L, respectively, according to the diagnostic criteria of the US National Cholesterol Education Program Adult Treatment Panel III (NCEPIII)^43^ and Joint Committee for Developing Chinese Guidelines on Prevention and Treatment of Dyslipidemia in Adults (JCDCG)^44^.

### Sample size calculation

Hypothesizing an effect size of 0.25 for a power of 90% at p alpha < 0.05, for the three groups (no to mild OSA, moderate OSA, and severe OSA group), a power analysis showed that a sample size of at least 18 subjects per study group was required. The power analysis of the sample-size calculation was performed by using G*power software v. 3.1.9.2^45^.

### Bootstrapping analysis

A bootstrap analysis involves repeatedly sampling a fixed number of times from an observed dataset with replacement^46^. We derived the sampling distribution of the desired statistic from each sampled unit. The distribution of these individual statistics provided a framework to estimate the overall mean value. Then we conducted a simulation analysis to derive precise estimates from our data using the following steps:

1. Resampling with the replacement of items in the original dataset.
2. Calculation of the mean and standard deviation (SD) in the AHI variables, obesity indices, and the insulin, glucose, and lipid profiles in the three OSA severity groups using a simulation technique.
3. Performance of a repeated ANOVA followed by an ANCOVA and determination of the p values.
4. Repeat steps 1–3 2,000 times.

The simulation procedure for each scale is illustrated as a flow chart in Supplementary Fig. 4.

### Statistical analysis

Data are presented as means ± SD, medians (interquartile range), and numbers (percentage) if they were normally distributed, skewed, or categorical, respectively. Differences in sleep parameters, anthropometric variables, and lipids between groups were examined in an analysis of variance(ANOVA) or ANCOVA. The p values for linear trends across the three groups were calculated using the polynomial linear trend test for continuous variables and the linear-by-linear association test for dichotomous variables. Binary logistic regression analyses were used to determine risk factors for dyslipidemia. Univariate regression analysis was used to assess the correlation between each included variable and lipid profile, and if variables had a p < 0.1, a multivariate regression model was performed using the aforementioned variable. A p value < 0.05 was considered to indicate statistical significance.

## Additional Information

**How to cite this article**: Xia, Y. *et al*. Prevalence and Predictors of Atherogenic Serum Lipoprotein Dyslipidemia in Women with Obstructive Sleep Apnea. *Sci. Rep.* **7**, 41687; doi: 10.1038/srep41687 (2017).

**Publisher's note:** Springer Nature remains neutral with regard to jurisdictional claims in published maps and institutional affiliations.

## Supplementary Material

Supplementary Tables and Figures

## Figures and Tables

**Table 1:** Characteristics and Serum Lipid Levels in women according to OSA severity.

	No to mild OSA (n = 341)	Moderate OSA (n = 95)	Severe OSA (n = 134)	P	P for trend
**Characteristics**					
Age (yrs)	44(36–53)	54(47–60)	55(47–60)	<0.01	<0.01
BMI(kg/m^2^)	23.6 ± 3.3	25.4 ± 3.3	28.2 ± 4.5	<0.01	<0.01
NC (cm)	34.0 ± 2.7	35.6 ± 2.6	37.4 ± 2.9	<0.01	<0.01
WC (cm)	83.8 ± 9.2	89.6 ± 8.5	96.0 ± 11.4	<0.01	<0.01
HC (cm)	95.0 ± 6.6	98.6 ± 6.2	102.5 ± 9.0	<0.01	<0.01
WHR	0.88 ± 0.07	0.91 ± 0.06	0.94 ± 0.06	<0.01	<0.01
AHI	2.2(0.5–7.0)	22.0(19.3–25.3)	56.0(39.1–74.1)	<0.01	<0.01
LSpO_2_	92.0(88.0–95.0)	81.0(74.0–86.0)	73.0(62.0–81.0)	<0.01	<0.01
ODI	3.5(0.5–7.7)	25.4(20.3–31.3)	61.6(45.6–80.5)	<0.01	<0.01
MAI	10.8(6.1–20.5)	18.8(7.7–27.0)	30.6(9.1–49.1)	<0.01	<0.01
Glucose	4.9(4.6–5.3)	5.4(5.0–6.0)	5.5(5.0–6.1)	<0.01	<0.01
Insulin	7.3(4.9–10.3)	10.7(7.2–15.6)	12.82(9.33–19.62)	<0.01	<0.01
HOMA-IR	1.6(1.0–2.4)	2.6(1.8–4.1)	3.1(2.1–5.1)	<0.01	<0.01
**Serum lipid levels**					
TC	4.60 ± 0.99	5.00 ± 0.91	4.98 ± 0.92	0.22	0.18
TG	1.00(0.70–1.49)	1.40(0.99–1.96)	1.56(1.08–2.22)	0.07	0.21
HDL-C	1.26 ± 0.27	1.26 ± 0.28	1.19 ± 0.25	0.51	0.59
LDL-C	2.82 ± 0.88	3.12 ± 0.80	3.13 ± 0.81	0.30	0.20
apoA-I	1.18 ± 0.20	1.19 ± 0.19	1.16 ± 0.21	0.81	0.52
apoB	0.76 ± 0.18	0.84 ± 0.15	0.87 ± 0.16	0.09	0.04
apoE	4.18(3.39–5.02)	4.66(3.98–5.31)	4.68(3.70–5.85)	0.60	0.54
Lp(a)	9.30(5.14–19.30)	9.10(5.30–13.50)	10.40(5.68–20.00)	0.04	0.40

Abbreviations: OSA, obstructive sleep apnea; BMI, Body mass index; NC, neck circumference; WC, waist circumference, HC, hip circumference; WHR, waist to hip ratio; HOMA-IR, insulin resistance index calculated by the homeostasis model assessment; AHI, apnea-hypopnea index;LSpO_2_, lowest oxygen saturation; ODI, oxygen desaturation index; MAI, microarousal index;TC, total cholesterol; TG, triglycerides; HDL-C, high-density lipoprotein cholesterol; LDL-C, low-density lipoprotein cholesterol; apo, apolipoprotein; Lp(a), lipoprotein(a).

Differences of characteristics among three groups were examined by using ANOVA. Differences of serum lipid levels were examined by using ANCOVA, with P values adjusted for age, BMI, WHR, insulin and glucose. P for trend was tested using the polynomial linear trend test for continuous variables.

**Table 2:** Prevalence of Lipid Abnormalities in women according to OSA severity.

	No to mild OSA (n = 341)	Moderate OSA (n = 95)	Severe OSA (n = 134)	P for trend
**Percentages of lipid abnormalities**				
TC	87(25.5)	36(37.9)	56(41.8)	<0.01
TG	57(16.7)	34(35.8)	56(41.8)	<0.01
HDL-C	69(20.2)	21(22.1)	35(26.1)	0.17
LDL-C	84(24.6)	35(36.8)	53(39.6)	<0.01
apoA-I	175(51.3)	55(57.9)	81(60.4)	0.06
apoB	12(3.5)	2(2.1)	13(9.7)	0.01
apoE	128(37.5)	40(42.1)	72(53.7)	<0.01
Lp (a)	41(12.0)	6(6.3)	11(8.2)	0.14

Abbreviations: OSA, obstructive sleep apnea; TC, total cholesterol; TG, triglycerides; HDL-C, high-density lipoprotein cholesterol; LDL-C, low-density lipoprotein cholesterol; apo, apolipoprotein; Lp(a), lipoprotein(a). P for trend was estimated by the linear-by linear association test for dichotomous variables.

**Table 3:** Binary logistic regression model of selected factors and dyslipidemia in women.

	β	OR(95% CI)	P
age	0.026	1.03(1.01,1.04)	<0.01
WHR	0.588	1.80(1.31,2.47)	<0.01
ODI	0.019	1.02(1.01,1.03)	<0.01

Abbreviations: OR, odd ratio; CI, confidential interval; WHR, waist to hip ratio; ODI, oxygen desaturation index. We performed Forward Binary Logistic regression. The variables added in binary regression model included age, body mass index(BMI), WHR, insulin, glucose, apnea-hypopnea index(AHI), ODI, lowest oxygen saturation(LSpO_2_) and micoarousal index(MAI).

**Table 4:** Multivariate linear regression model of selected factors and lipid profile in women.

	TC		TG		HDL-C		LDL-C		ApoA-I		ApoB		ApoE	
	β	p	Β	p	β	p	β	P	β	p	β	p	β	p
Age (5-year increase)	0.096	<0.01	0.072	<0.01	−	−	0.057	<0.01	0.019	<0.01	0.017	<0.01	0.083	<0.01
BMI (2-kg/m^2^ increase)	-0.018	0.47	0.018	0.48	−0.015	0.03	-0.005	0.81	−0.010	0.03	−0.003	0.48	−0.010	0.80
WHR (0.1-unit increase)	0.137	0.03	0.165	<0.01	-0.011	0.54	0.093	0.10	−	−	0.033	<0.01	0.236	0.02
Insulin (5-μU/mLincrease)	−0.019	0.58	0.145	<0.01	−0.034	<0.01	−0.017	0.58	−0.010	0.13	0.010	0.09	0.161	<0.01
Glucose (0.5-mmol/L increase)	0.078	<0.01	0.029	0.27	−0.003	0.74	0.081	<0.01	−	−	0.019	<0.01	0.001	0.98
AHI (per 5 unit increase)	0.003	0.87	0.002	0.91	−0.006	0.32	0.008	0.65	−	−	0.003	0.38	0.029	0.33
ODI (per 5 unit increase)	−0.007	0.70	0.026	0.14	0.006	0.21	−0.009	0.58	−	−	−0.004	0.16	−0.016	0.55
MAI (per 5 unit increase)	−	−	−0.011	0.33	−	−	0.013	0.22	−	−	−	−	−	−
LSpO_2_ (per 5 unit decrease)	0.033	0.22	−0.042	0.12	0.003	0.73	0.017	0.48	−	−	0.011	0.02	−0.007	0.86

Abbreviations: TC, total cholesterol; TG, triglycerides; HDL-C, high-density lipoprotein cholesterol; LDL-C, low-density lipoprotein cholesterol; apo, apolipoprotein; Lp(a), lipoprotein(a); BMI, Body mass index; WHR, waist to hip ratio; AHI, apnea-hypopnea index; ODI, oxygen desaturation index; MAI, microarousal index; LSpO_2_, lowest oxygen saturation; −, the variable was not included in the multivariate regression model.

The values of β for continuous variables mean β for an increase unit, β for WHR, an increase of 0.1 WHR units.
