# Supplementary material for: Prevalence and Predictors of Atherogenic Serum Lipoprotein Dyslipidemia in Women with Obstructive Sleep Apnea
Source: Sci Rep. 2017 Jan 30;7:41687. doi: 10.1038/srep41687 (PMC5278373; doi:10.1038/srep41687)
Supplement: Supplementary Tables and Figures [file srep41687-s1.pdf]

Prevalence and Predictors of Atherogenic Serum Lipoprotein Dyslipidemia in Women with Obstructive Sleep Apnea

Yunyan Xia[1,2,3,*],MD, Yiqun Fu[1,2,3,*],MD, Yuyu Wang[1,2,3] ,MD, Yingjun Qian[1,2,3], MD, Xinyi Li[1,2,3], MD, Huajun Xu[1,2,3],MD,Jianyin Zou[1,2,3],PhD,Jian Guan[1,2,3], MD, PhD, Hongliang Yi[1,2,3], MD, PhD, Lili Meng, MD[1,2,3], MD, Xulan Tang[1,2,3], MD, Huaming Zhu[1,2,3], MD, Dongzhen Yu[1,2,3], MD, PhD, Huiqun Zhou[1,2,3], MD, Kaiming Su[1,2,3], MD,PhD, and Shankai Yin[1,2,3], MD, PhD

1 Department of Otolaryngology Head and Neck Surgery& Center of Sleep Medicine, Shanghai Jiao Tong University Affiliated Sixth People's Hospital, Yishan Road 600, Shanghai, 200233, China.

2 Otolaryngological Institute of Shanghai Jiao Tong University, Yishan Road 600, Shanghai, 200233, China.

3 Clinical Research Center, Shanghai Jiao Tong University School of Medicine, South Chongqing Road 225, Shanghai,200020, China.

*these authors contributed equally to this paper.

Corresponding author: Huajun Xu, MD(E-mail:sunnydayxu2010@163.com), Jian Guan, MD, PhD (E-mail: guanjian0606@sina.com) and Shankai Yin, MD, PhD (E-mail: skyin@sjtu.edu.cn

Supplementary Table S1 Serum Lipid Levels in Women according to OSA severity Stratified by obesity or age

	Non-obese women (n=450)					obese women(n=120)				
	No to mild OSA (n=301)	Moderate OSA (n=76)	Severe OSA (n=73)	P	P for trend	No to mild OSA (n=40)	Moderate OSA (n=19)	Severe OSA (n=61)	P	P for trend
**Serum lipid levels**										
TC	4.59±1.00	5.04±0.95	5.01±0.98	0.47	0.40	4.65±0.96	4.81±0.72	4.94±0.85	0.43	0.20
TG	0.97(0.69-1.42)	1.35(0.95-1.86)	1.61(1.06-2.26)	0.31	0.17	1.44(0.85-1.82)	1.70(1.37-2.17)	1.50(1.09-2.00)	0.14	0.35
HDL-C	1.26±0.28	1.27±0.28	1.23±0.27	0.32	0.37	1.21±0.25	1.23±0.31	1.16±0.23	0.51	0.37
LDL-C	2.82±0.90	3.20±0.81	3.10±0.82	0.46	0.69	2.85±0.75	2.83±0.73	3.18±0.79	0.18	0.10
apoA-I	1.18±0.20	1.20±0.18	1.18±0.21	0.99	0.89	1.19±0.20	1.17±0.20	1.14±0.21	0.58	0.30
apoB	0.75±0.18	0.84±0.16	0.86±0.18	0.40	0.19	0.77±0.18	0.83±0.12	0.87±0.15	0.09	**0.03**
apoE	4.16(3.37-4.96)	4.62(3.91-5.28)	4.55(3.48-5.90)	0.85	0.65	4.43(3.58-5.55)	5.07(4.20-5.43)	5.06(3.77-5.79)	0.66	0.51
Lp(a)	9.50(5.15-19.30)	9.06(5.05-13.30)	10.20(5.30-20.40)	0.06	0.35	7.72(5.04-20.10)	9.16(5.50-13.70)	11.10(6.00-18.70)	0.76	0.88
	Age＜55 women (n=382)					Age≥55 women(n=188)				
	No to mild OSA (n=268)	Moderate OSA (n=48)	Severe OSA (n=66)	P	P for trend	No to mild OSA (n=73)	Moderate OSA (n=47)	Severe OSA (n=68)	P	P for trend
**Serum lipid levels**										
TC	4.42±0.92	4.69±0.80	4.90±0.93	0.07	0.03	5.24±1.00	5.31±0.91	5.06±0.92	0.50	0.35
TG	0.91(0.66-1.38)	1.40(0.95-1.81)	1.51(1.04-2.22)	0.30	0.32	1.32(0.98-1.80)	1.44(1.16-2.28)	1.61(1.17-2.23)	0.35	0.48
HDL-C	1.25±0.27	1.24±0.29	1.18±0.26	0.09	0.05	1.29±0.28	1.28±0.28	1.20±0.25	0.18	0.09
LDL-C	2.71±0.84	2.98±0.73	3.10±0.79	0.18	0.08	3.24±0.90	3.27±0.85	3.16±0.82	0.86	0.66
apoA-I	1.15±0.20	1.14±0.16	1.14±0.20	0.46	0.21	1.29±0.21	1.24±0.20	1.19±0.22	0.03	0.01
apoB	0.72±0.17	0.79±0.16	0.85±0.17	0.04	0.01	0.87±0.18	0.89±0.13	0.88±0.16	0.99	0.97
apoE	4.05(3.36-4.78)	4.59(3.76-5.05)	4.59(3.66-5.76)	0.74	0.46	4.85(3.59-5.81)	4.93(4.10-5.60)	5.10(3.71-5.92)	0.82	0.97
Lp(a)	9.10(4.93-18.89)	7.80(5.00-12.43)	10.43(5.38-16.18)	0.10	0.54	10.00(5.90-21.95)	9.90(6.40-17.70)	10.40(6.20-21.13)	0.23	0.43

Obesity was defined as a body mass index≥28 kg/m^2. Age<55 was defined as age less than 55 years old, age≥55 was defined as no less than 55 years old. Normally distributed data were presented as means ± standard deviation (SD), skewed data were presented as the median (interquartile range).

Abbreviations: OSA, obstructive sleep apnea; TC, total cholesterol; TG, triglycerides; HDL-C, high-density lipoprotein cholesterol; LDL-C, low-density lipoprotein cholesterol; apo, apolipoprotein; Lp(a), lipoprotein(a).Difference of serum lipid levels were examined by using ANCOVA. P values were adjusted for age, BMI, WHR, insulin and glucose. P for trend was tested using the polynomial linear trend test for continuous variable.

Supplementary Table S2 Prevalence of Lipid Abnormalities in Women according to OSA severity Stratified by obesity or age

	Non-obese women (n=450)				obese women(n=120)			
	No to mild OSA (n=301)	Moderate OSA (n=76)	Severe OSA (n=73)	P for trend	No to mild OSA (n=40)	Moderate OSA (n=19)	Severe OSA (n=61)	P for trend
**Percentages of lipid abnormalities**								
TC	78(25.9)	29(38.2)	32(43.8)	＜0.01	9(22.5)	7(36.8)	24(39.3)	0.09
TG	44(14.6)	24(31.6)	32(43.8)	＜0.01	13(32.5)	10(52.6)	24(39.3)	0.57
HDL-C	58(19.3)	17(22.4)	18(24.7)	0.28	11(27.5)	4(21.1)	17(27.9)	0.93
LDL-C	76(25.2)	30(39.5)	31(42.5)	＜0.01	8(20.0)	5(26.3)	22(36.1)	0.08
apoA-I	154(51.2)	42(55.3)	41(56.2)	0.38	21(52.5)	213(68.4)	40(65.6)	0.21
apoB	11(3.7)	2(2.6)	7(9.6)	0.06	1(2.5)	0(0)	6(9.8)	0.10
apoE	108(35.9)	28(36.8)	35(47.9)	0.08	20(50.0)	12(63.2)	37(60.7)	0.32
Lp (a)	36(12.0)	5(8.2.6)	6(0)	0.21	5(12.5)	1(5.3)	11(9.2)	0.51
	Age＜55 women (n=382)				Age≥55 women(n=188)			
	No to mild OSA (n=268)	Moderate OSA (n=48)	Severe OSA (n=66)	P for trend	No to mild OSA (n=73)	Moderate OSA (n=47)	Severe OSA (n=68)	P for trend
**Percentages of lipid abnormalities**								
TC	47(17.5)	11(22.9)	25(37.9)	＜0.01	40(54.8)	25(53.2)	31(51.1)	0.28
TG	36(13.4)	17(35.4)	26(39.4)	＜0.01	21(28.8)	17(36.2)	30(44.1)	0.06
HDL-C	56(20.9)	12(25.0)	21(31.8)	0.06	13(17.8)	9(19.1)	14(20.6)	0.68
LDL-C	55(20.5)	14(29.2)	24(36.4)	＜0.01	29(39.7)	21(44.7)	29(42.6)	0.72
apoA-I	153(57.1)	35(72.9)	39(59.1)	0.42	22(30.1)	20(42.6)	42(61.8)	＜0.01
apoB	6(2.2)	2(4.1)	7(10.6)	＜0.01	6(8.2)	0(0)	6(8.8)	0.91
apoE	90(33.6)	16(33.3)	31(47.0)	0.06	38(52.1)	24(51.1)	41(60.3)	0.33
Lp (a)	30(11.2)	2(4.2)	5(7.6)	0.22	11(15.1)	4(8.5)	6(8.8)	0.24

Obesity was defined as a body mass index ≥28 kg/m^2. Age<55 was defined as age less than 55 years old, age≥55 was defined as no less than 55 years old. Categorical data were presented as the number (percentage).

Abbreviations: OSA, obstructive sleep apnea; TC, total cholesterol; TG, triglycerides; HDL-C, high-density lipoprotein cholesterol; LDL-C, low-density lipoprotein cholesterol; apo, apolipoprotein; Lp(a), lipoprotein(a). P for trend was estimated by the linear-by linear association test for dichotomous variables.

Supplementary Table S3 Serum Lipid Levels in women according to OSA severity

	No to mild OSA (n=367)	Moderate OSA (n=105)	Severe OSA (n=98)	P	P for trend
**Serum lipid levels**					
TC	4.61±0.98	5.08±0.95	4.92±0.92	0.13	0.39
TG	1.02(0.71-1.52)	1.39(0.99-1.87)	1.60(1.16-2.26)	0.44	0.35
HDL-C	1.25±0.28	1.28±0.24	1.16±0.24	0.08	0.88
LDL-C	2.83±0.86	3.21±0.82	3.08±0.82	0.15	0.44
apoA-I	1.18±0.20	1.21±0.20	1.14±0.21	0.28	0.27
apoB	0.76±0.18	0.86±0.16	0.86±0.17	0.10	0.18
apoE	4.22(3.41-5.05)	4.59(3.75-5.45)	4.95(3.83-5.86)	0.60	0.32
Lp(a)	9.40(5.20-19.04)	9.50(5.75-16.45)	10.20(5.38-17.00)	0.34	0.28

Abbreviations: OSA, obstructive sleep apnea; TC, total cholesterol; TG, triglycerides; HDL-C, high-density lipoprotein cholesterol; LDL-C, low-density lipoprotein cholesterol; apo, apolipoprotein; Lp(a), lipoprotein(a). Differences of serum lipid levels were examined by using ANCOVA, with P values adjusted for age, body-mass index(BMI), waist-to-hip ratio(WHR), insulin and glucose. P for trend was tested using the polynomial linear trend test for continuous variables.

Note: To figure out if the associations between the lipid levels and the sleep parameters would change when we used a higher cut-off, we defined that apnea-hypopnea index(AHI) of ＜10, ⩾10, ⩾20 and ⩾40 event per hour represented no, mild, moderate and severe obstructive

sleep apnea(OSA) respectively. We found that, after fully adjusted for age, BMI and WHR, insulin level and glucose level, no significant differences in serum lipid levels were seen among the three groups. No significant increased trends in serum lipid levels were seen in women with increasing OSA severity.

Supplementary Table S4 Prevalence of Lipid Abnormalities in women according to OSA severity

	No to mild OSA (n=367)	Moderate OSA (n=105)	Severe OSA (n=98)	P for trend
**Percentages of lipid abnormalities**				
TC	94(25.6)	45(42.9)	40(40.8)	＜0.01
TG	67(18.3)	37(35.2)	43(43.9)	＜0.01
HDL-C	75(20.4)	20(19.0)	30(30.6)	0.07
LDL-C	90(24.5)	46(43.8)	36(36.7)	＜0.01
apoA-I	191(52.0)	58(55.2)	62(63.3)	0.05
apoB	12(3.3)	6(5.7)	9(9.2)	0.01
apoE	138(37.6)	47(44.8)	55(56.1)	＜0.01
Lp (a)	42(11.4)	10(9.5)	6(6.1)	0.12

Abbreviations: OSA, obstructive sleep apnea; TC, total cholesterol; TG, triglycerides; HDL-C, high-density lipoprotein cholesterol; LDL-C, low-density lipoprotein cholesterol; apo, apolipoprotein; Lp(a), lipoprotein(a). P for trend was estimated by the linear-by linear association test for dichotomous variables.

Note: To figure out if the associations between the lipid levels and the sleep parameters would change when we used a higher cut-off, we defined that apnea-hypopnea index(AHI) of ＜10, ⩾10, ⩾20 and ⩾40 event per hour represented no, mild, moderate and severe obstructive sleep apnea(OSA) respectively. We found that the prevalance of dyslipidemia in TC, TG, LDL-C, apoB and apoE increased with increasing

OSA severity.

Supplementary Table S5 Univariate regression analysis model of selected factors and lipid profile in women

	TC		TG		HDL-C		LDL-C		apoA-I		apoB		apoE		LP(a)	
	β	p	β	p	β	p	β	p	β	p	β	P	β	p	β	p
Age (5-yearincrease)	0.125	＜0.01	0.110	＜0.01	0.001	0.91	0.084	＜0.01	0.016	＜0.01	0.026	＜0.01	0.121	＜0.01	0.081	0.74
BMI(2-kg/m^2 increase)	0.060	＜0.01	0.131	＜0.01	-0.026	＜0.01	0.054	＜0.01	-0.009	0.04	0.019	＜0.01	0.116	＜0.01	-0.063	0.83
WHR(0.1-unit increase)	0.285	＜0.01	0.393	＜0.01	-0.046	＜0.01	0.213	＜0.01	0.008	0.51	0.072	＜0.01	0.428	＜0.01	0.772	0.36
Insulin(5-μU/mL increase)	0.061	0.04	0.226	＜0.01	-0.043	＜0.01	0.059	0.02	-0.014	0.02	0.029	＜0.01	0.221	＜0.01	0.067	0.88
Glucose(0.5-mmol/L increase)	0.127	＜0.01	0.134	＜0.01	-0.018	0.01	0.115	＜0.01	0.000	0.92	0.034	＜0.01	0.114	＜0.01	0.293	0.43
AHI(per 5 unit increase)	0.032	＜0.01	0.054	＜0.01	-0.006	＜0.01	0.028	＜0.01	-0.003	0.13	0.009	＜0.01	0.053	＜0.01	-0.124	0.34
ODI(per 5 unit increase)	0.027	＜0.01	0.051	＜0.01	-0.005	0.02	0.023	＜0.01	-0.002	0.23	0.008	＜0.01	0.044	＜0.01	-0.130	0.25
MAI(per 5 unit increase)	0.014	0.20	0.028	0.02	0.003	0.40	0.023	0.02	-0.003	0.22	0.003	0.10	0.023	0.17	0.046	0.78
$LSpO_2$ (per 5 unit decrease)	0.078	＜0.01	0.095	＜0.01	-0.012	0.01	0.060	＜0.01	-0.002	0.56	0.022	＜0.01	0.099	＜0.01	-0.316	0.24

Abbreviations: TC, total cholesterol; TG, triglycerides; HDL-C, high-density lipoprotein cholesterol; LDL-C, low-density lipoprotein cholesterol; apo, apolipoprotein; Lp(a), lipoprotein(a); BMI, Body mass index; WHR, waist to hip ratio; AHI, apnea-hypopnea index; ODI, oxygen desaturation index; MAI, microarousal index; LSpO2, lowest oxygen saturation. The values of β for continuous variables represent β for an increase unit, β for WHR, an increase in 0.1 WHR units.

Supplementary Table S6 Binary logistic regression model of selected factors and dyslipidemia in age≤45 women

	β	OR(95%CI)	P
ODI	0.024	1.02(1.01,1.04)	＜0.01
WHR	0.687	1.99(1.28,3.10)	＜0.01

Abbreviations: OR, odd ratio; ODI, oxygen desaturation index; WHR, waist to hip ratio. We performed Forward Binary Logistic regression. The variables added in binary regression model included age, body mass index(BMI), WHR, insulin, glucose, apnea-hypopnea index(AHI), ODI, lowest oxygen saturation(LSpO2) and microarousal index(MAI).

Note: In age≤45 women, roughly premenopausal women, after adjusted for age, BMI, insulin, glucose, AHI, MAI and LSpO2, the major determinants affecting dyslipidemia were WHR and ODI (OR=1.99, p＜0.01; OR=1.02, p＜0.01, respectively).

Supplementary Table S7 Binary logistic regression model of selected factors and dyslipidemia in age⩾55 women

	β	OR(95%CI)	P
MAI	0.032	1.03(1.01,1.06)	$<$0.01

Abbreviations: OR, odd ratio; MAI, microarousal index. We performed Forward Binary Logistic regression. The variables added in binary regression model included age, body mass index(BMI), waist to hip ratio(WHR), insulin, glucose, apnea-hypopnea index(AHI), oxygen desaturation index(ODI), lowest oxygen saturation(LSpO2) and MAI.

Note: In age⩾55 women, roughly postmenopausal women, after adjusted for age, BMI, WHR, insulin, glucose, AHI, ODI and $LSpO_2$, the major independent determinants was MAI(OR=1.03, $p<0.01$).

Supplementary Figure 1 Histogram of p values for serum lipids from the repeated ANCOVA tests.

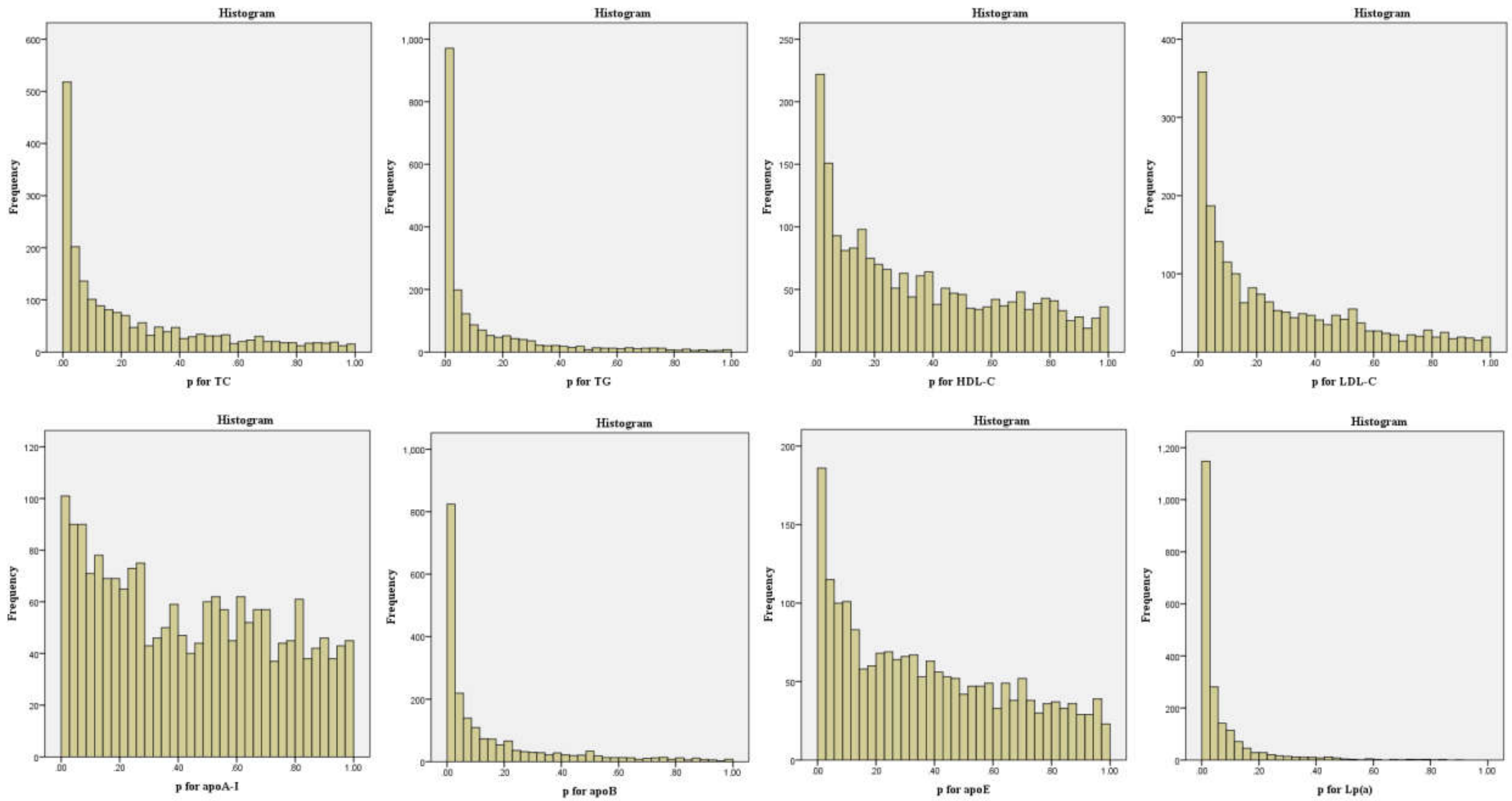

Abbreviations: ANCOVA, analysis of covariance; TC, total cholesterol; TG, triglycerides; HDL-C, high-density lipoprotein cholesterol; LDL-C, low-density lipoprotein cholesterol; apo, apolipoprotein; Lp(a), lipoprotein(a).

Supplementary Figure 2 Histogram of p values for serum lipids stratified by obesity from the repeated ANCOVA tests.

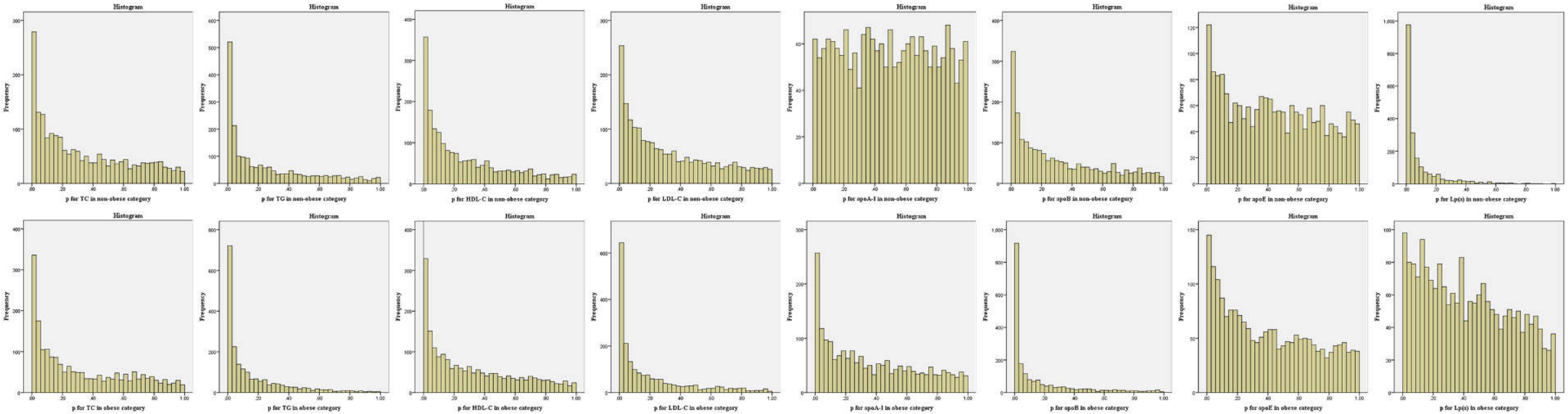

Abbreviations: ANCOVA, analysis of covariance; TC, total cholesterol; TG, triglycerides; HDL-C, high-density lipoprotein cholesterol; LDL-C, low-density lipoprotein cholesterol; apo, apolipoprotein; Lp(a), lipoprotein(a). Obesity was defined as a body mass index ⩾28 kg/m^2.

Supplementary Figure 3 Histogram of p values for serum lipids stratified by age from the repeated ANCOVA tests.

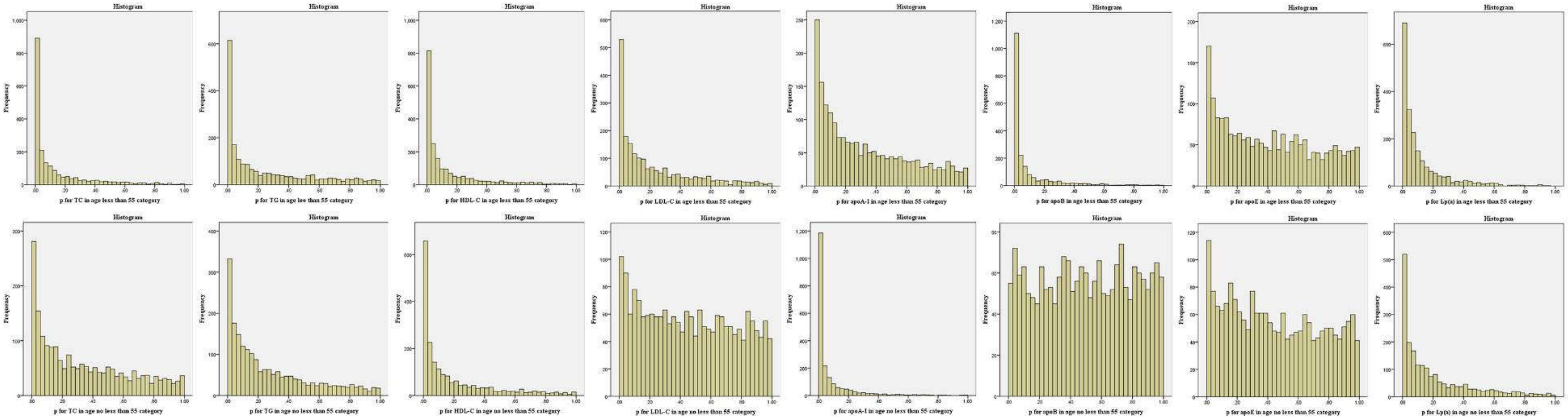

Abbreviations: ANCOVA, analysis of covariance; TC, total cholesterol; TG, triglycerides; HDL-C, high-density lipoprotein cholesterol; LDL-C, low-density lipoprotein cholesterol; apo, apolipoprotein; Lp(a), lipoprotein(a).

Supplementary Figure 4 Simulation procedure for the bootstrap analysis.

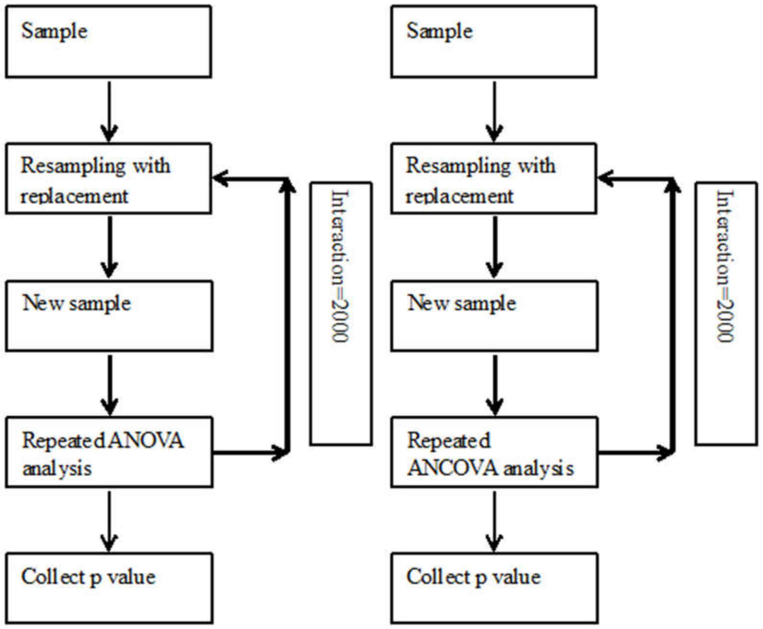

Figure 4a Figure 4b

Abbreviation: ANOVA, analysis of variance; ANCOVA, analysis of covariance.

Figure 4a Simulation ANOVA procedure for the bootstrap analysis. Figure 4b Simulation ANCOVA procedure for the bootstrap analysis.

Supplementary Figure 5 Histogram of p values for characteristics from the repeated ANOVA tests.

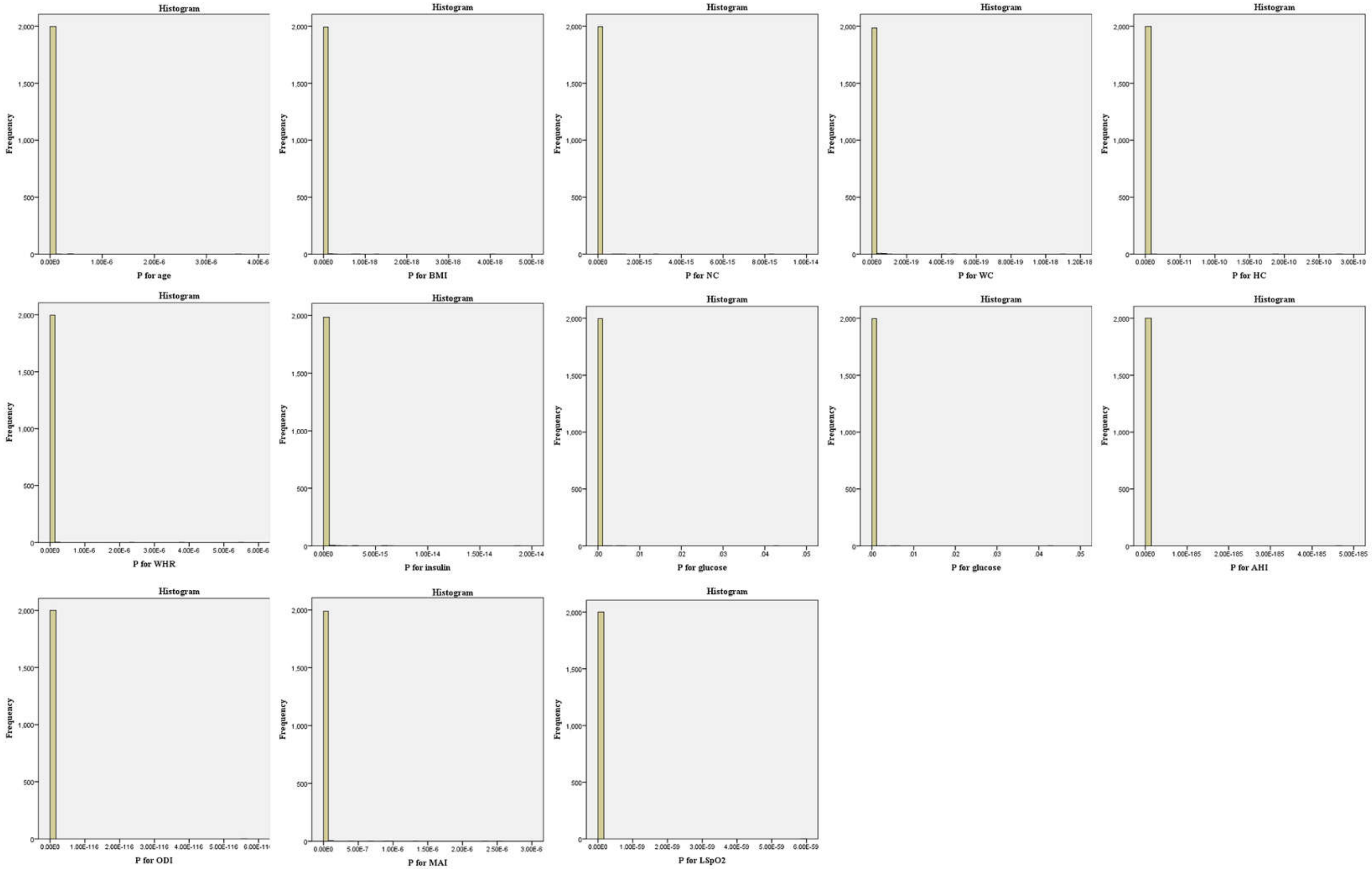

Abbreviations: ANOVA, analysis of variance; BMI, Body mass index; NC, neck circumference; WC, waist circumference, HC, hip circumference; WHR, waist to hip ratio; HOMA-IR, insulin resistance index calculated by the homeostasis model assessment; AHI, apnea-hypopnea index; LSpO2, lowest oxygen saturation; ODI, oxygen desaturation index; MAI, microarousal index.
